# Supplementary material for: The Associations of Iron Related Biomarkers with Risk, Clinical Severity and Mortality in SARS-CoV-2 Patients: A Meta-Analysis
Source: Nutrients. 2022 Aug 19;14(16):3406. doi: 10.3390/nu14163406 (PMC9416650; doi:10.3390/nu14163406)
Supplement: Supplementary file 1 [file nutrients-14-03406-s001.zip › Supplemental Figures.pdf]

(A)

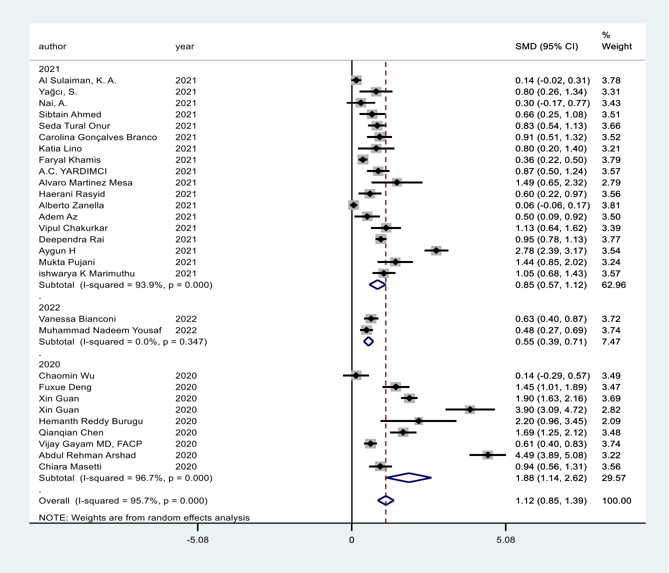

(B)

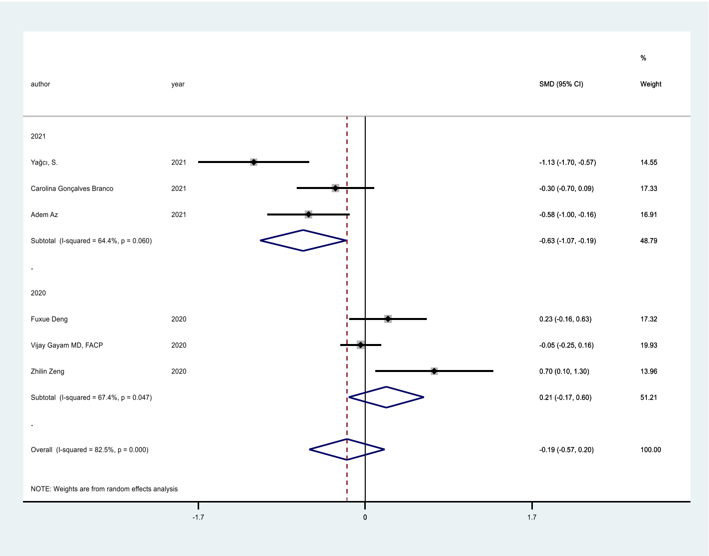

(C)

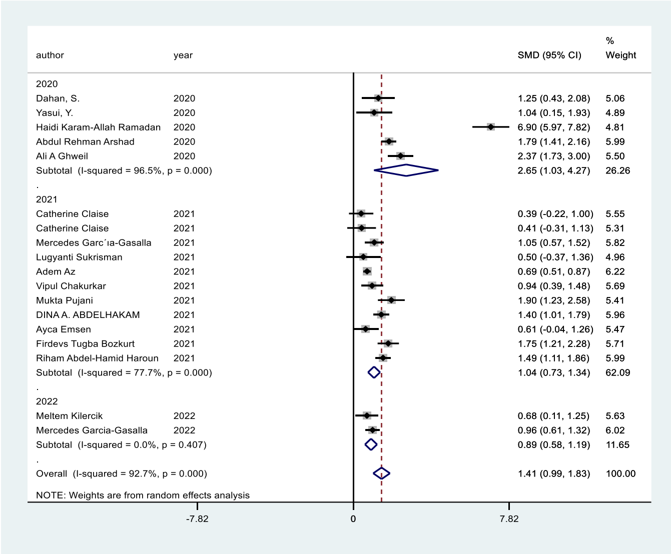

(D)

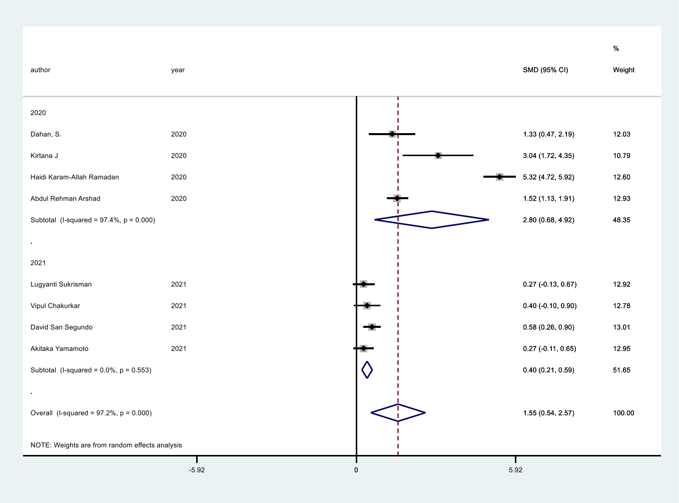

(E)

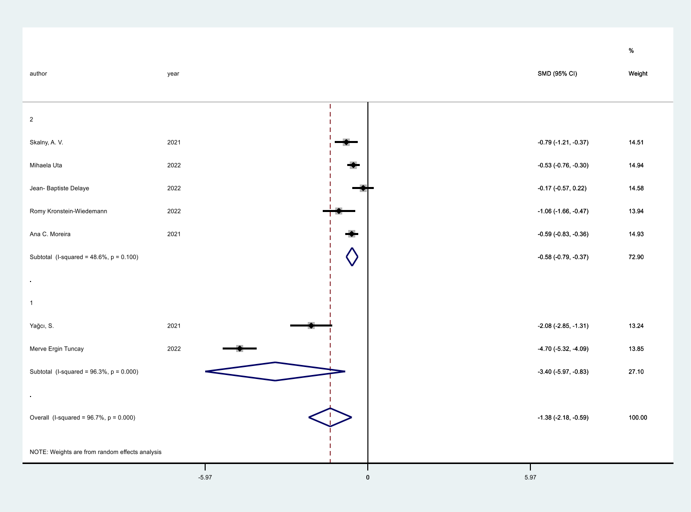

(F)

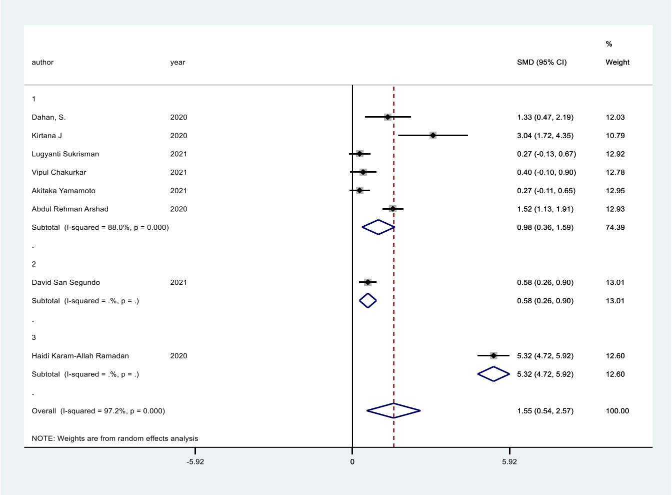

(G)

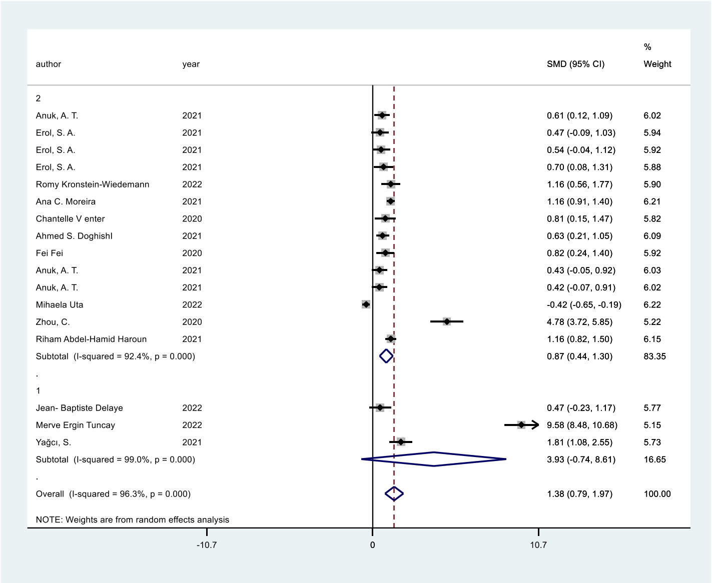

Supplemental Figure S1 Forest plot of standard mean difference (SMD) with corresponding 95% confidence interval (CI) of studies on subgroup analysis by publication year, continent and study type. (**A**) forest plot for mortality analysis about ferritin by publication year; (**B**) forest plot for mortality analysis about hemoglobin by publication year; (**C**) forest plot for severe-mild comparison about ferritin by publication year; (**D**) forest

plot for moderate-mild comparison about ferritin by publication year; (**E**) forest plot for risk analysis about serum iron by continent, “1” on behalf of Asia and “2” on behalf of Europe; (**F**) forest plot for moderate-mild comparison about ferritin by continent, “1” on behalf of Asia, “2” on behalf of Europe and “3” on behalf of Africa; (**G**) forest plot for risk analysis about ferritin by study type,“1” on behalf of cross-sectional study, “2” on behalf of case- control study and “3” on behalf of cohort study.

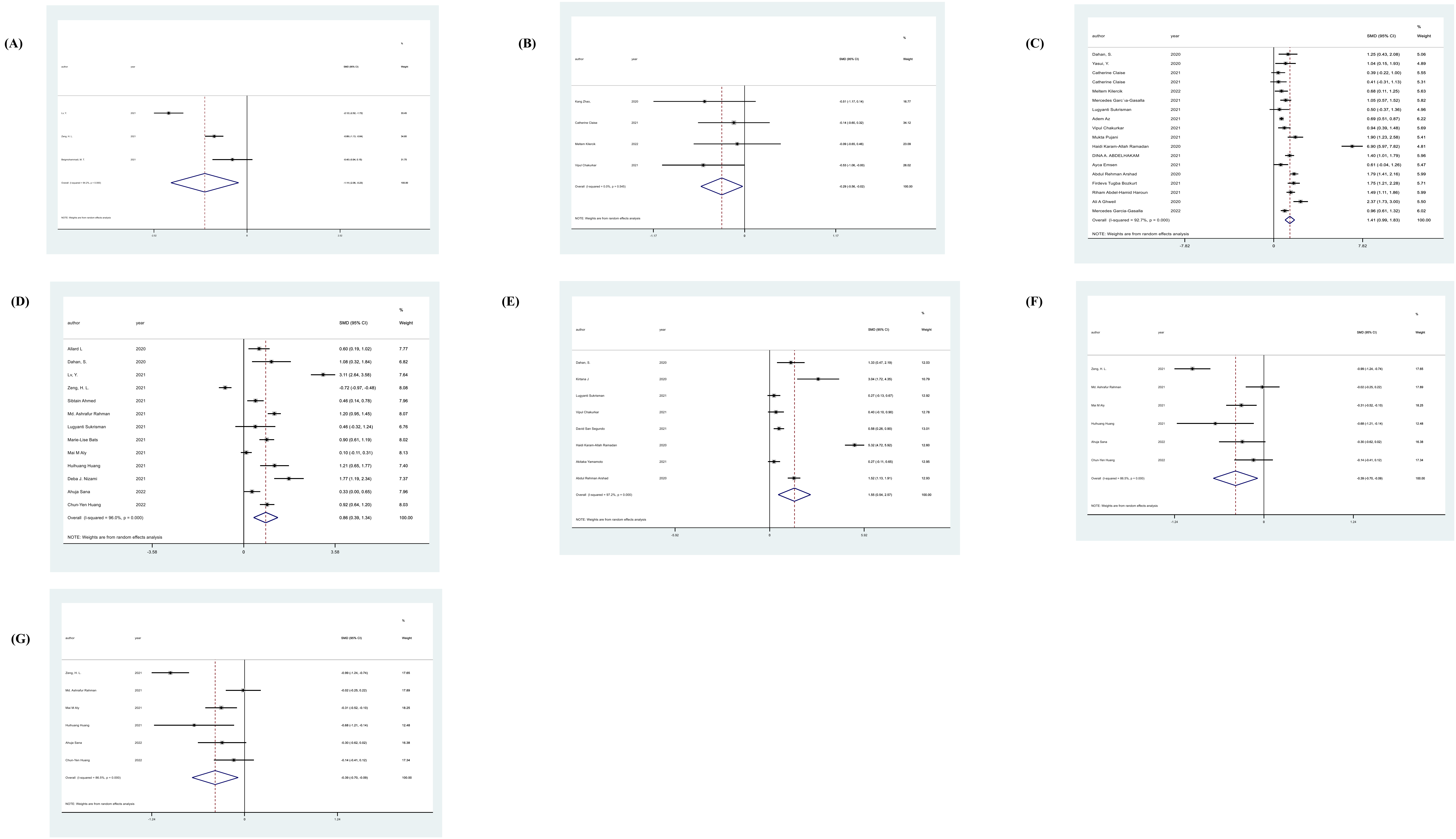

Supplemental Figure S2 Forest plot of standard mean difference (SMD) with corresponding 95% confidence interval (CI) of studies on iron-related biomarkers levels about severity meta-analysis. (**A**) Forest plot for severe-non severe comparison of serum iron; (**B**) forest plot for severe-mild comparison of serum iron; (**C**) for severe-mild comparison of ferritin; (**D**) for severe-non-severe comparison of ferritin; (**E**) for moderate-mild comparison of ferritin; (**F**) for severe-non severe comparison of hemoglobin; (G) for severe-mild comparison of hemoglobin.

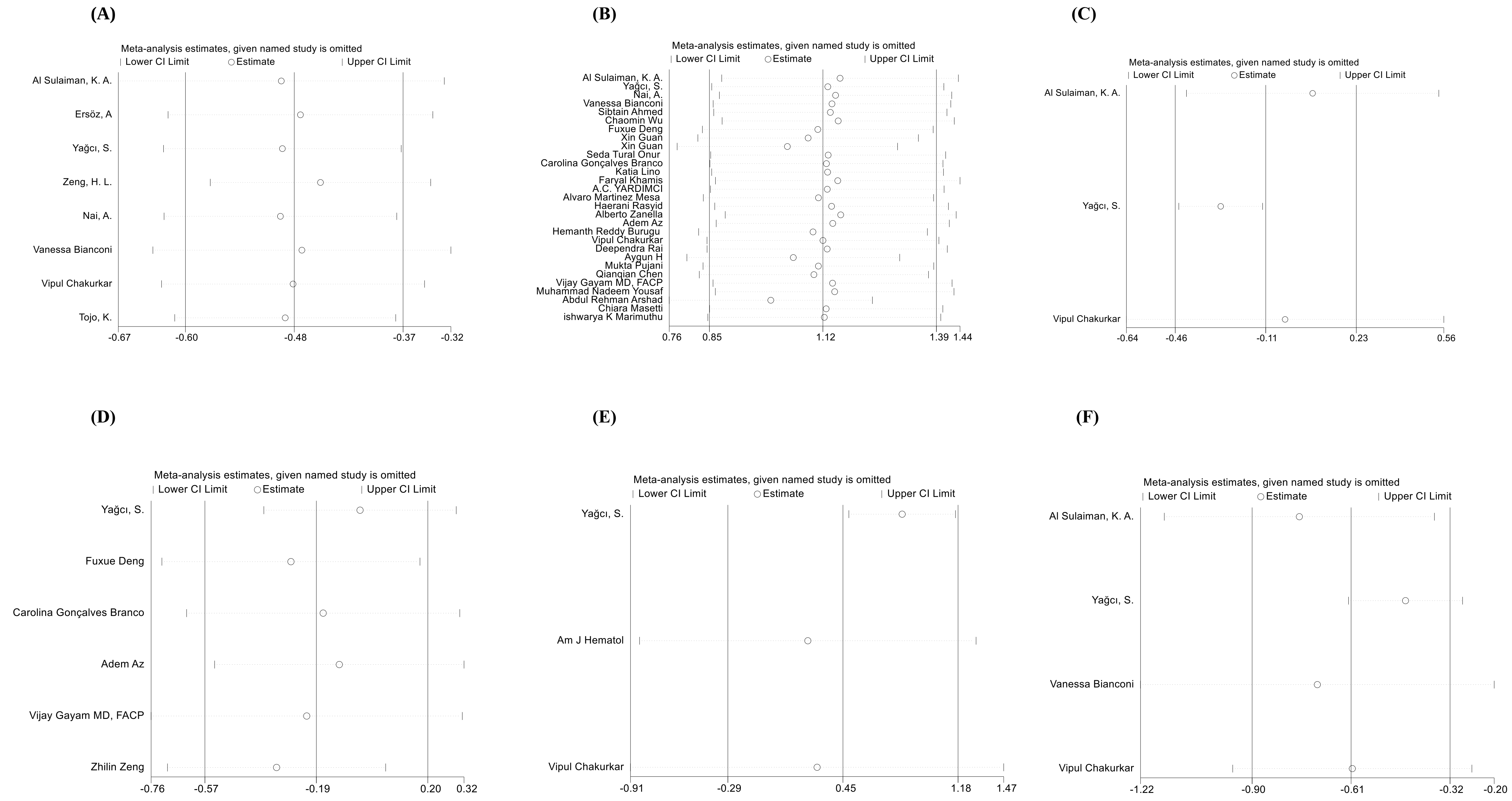

Supplemental Figure S3 The influence analysis results of iron-related biomarkers levels about mortality meta-analysis. (**A**) for serum iron; (**B**) for ferritin; (**C**) for TSAT; (**D**) for hemoglobin; (**E**) for hepcidin; (**F**) for TIBC.

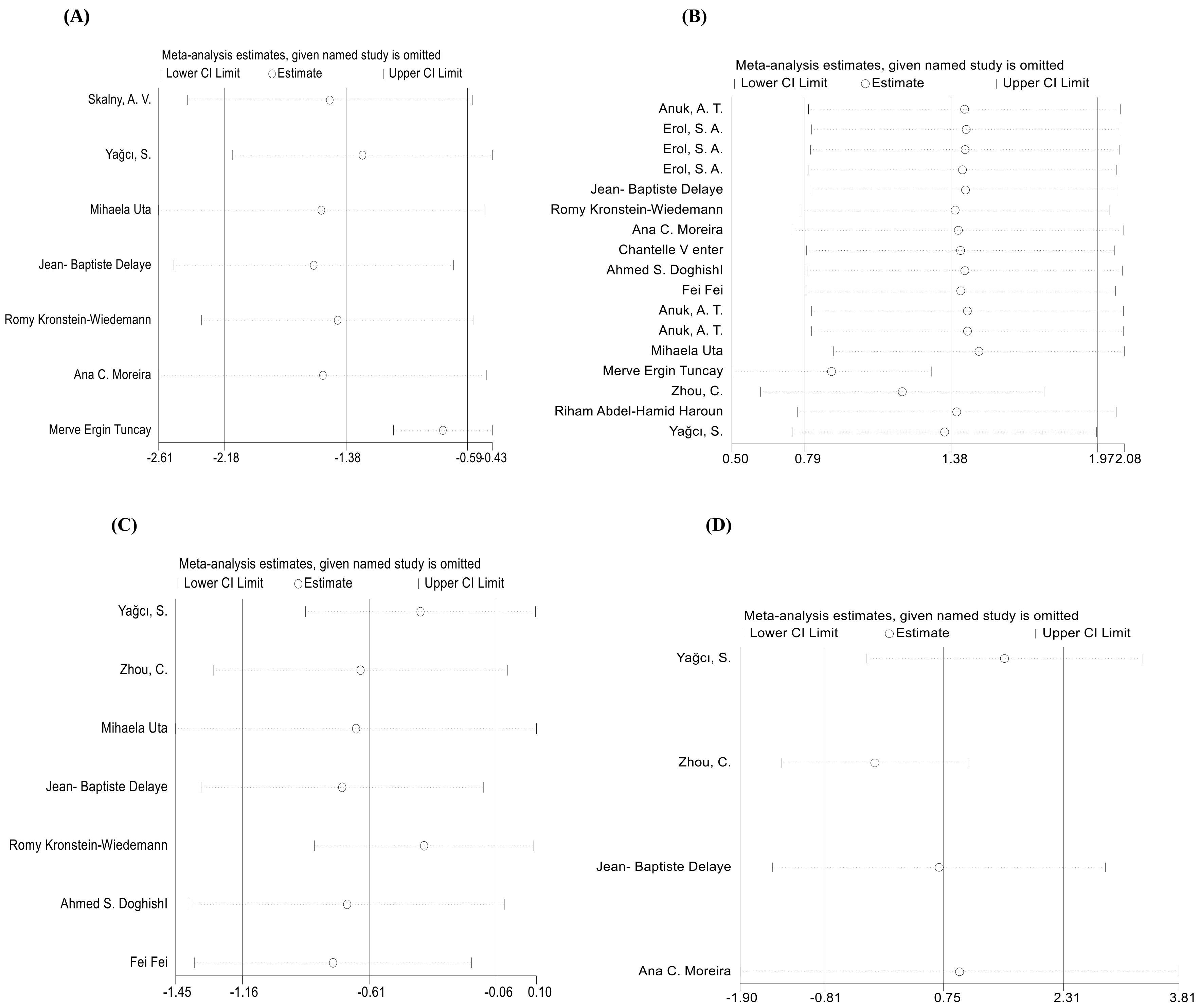

Supplemental Figure S4 The influence analysis results of iron-related biomarkers levels about risk meta-analysis. (**A**) for serum iron; (**B**) for ferritin; (**C**) for hemoglobin; (**D**) for hepcidin.

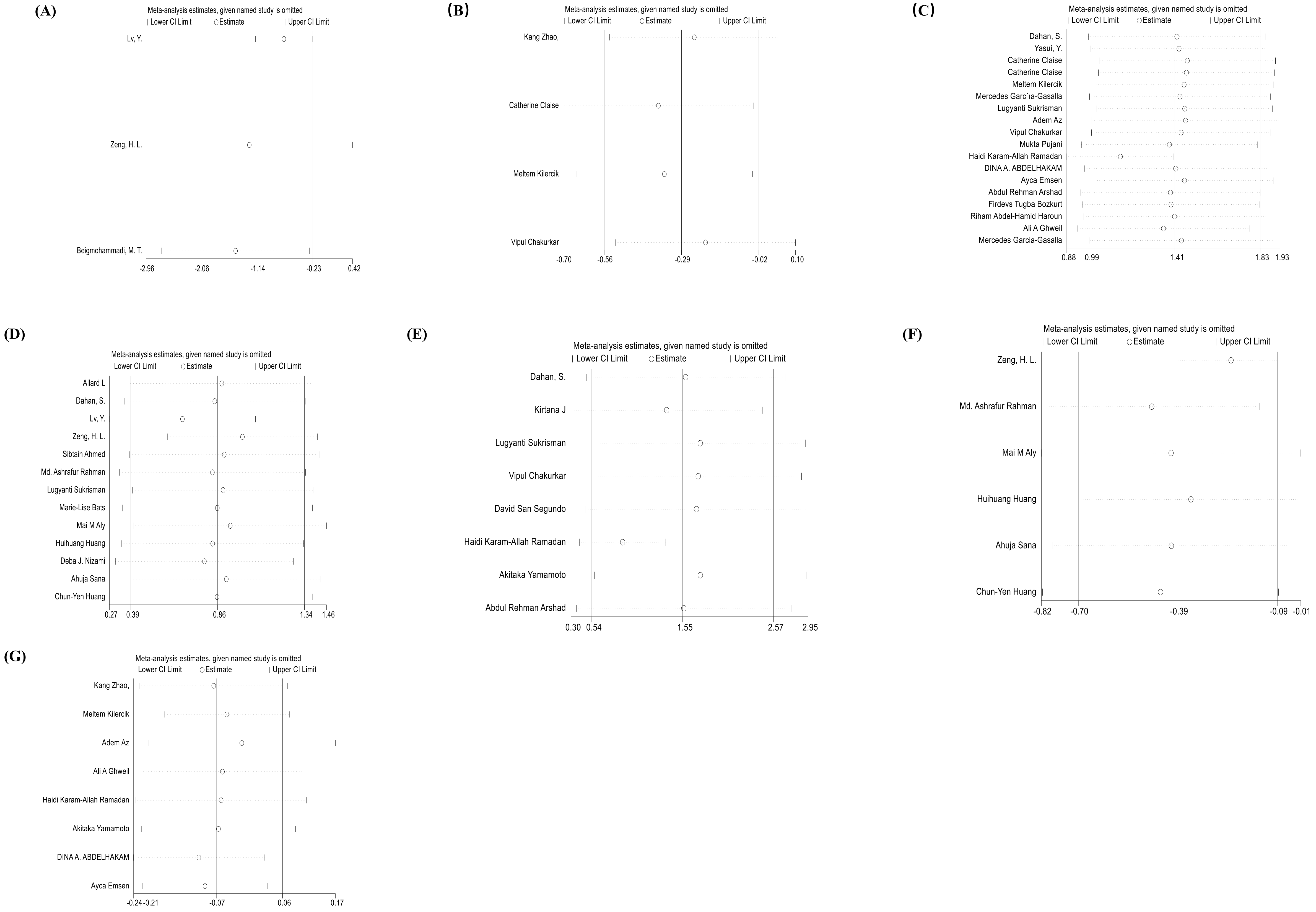

Supplemental Figure S5 The influence analysis results of iron-related biomarkers levels about severity meta-analysis. (**A**) for severe-non severe comparison of serum iron; (**B**) for severe-mild comparison of serum iron; (**C**) for severe-mild comparison of ferritin; (**D**) for severe-non severe comparison of ferritin; (**E**) for moderate-mild comparison of ferritin; (**F**) for severe-non severe comparison of hemoglobin; (**G**) for severe-mild comparison of hemoglobin.
