# Supplementary material for: The Associations of Iron Related Biomarkers with Risk, Clinical Severity and Mortality in SARS-CoV-2 Patients: A Meta-Analysis
Source: Nutrients. 2022 Aug 19;14(16):3406. doi: 10.3390/nu14163406 (PMC9416650; doi:10.3390/nu14163406)
Supplement: Supplementary file 1 [file nutrients-14-03406-s001.zip › Supplemental Table.pdf]

Table S1. Quality assessment of studies included in the meta-analysis.

Author (year)	Selection	Comparability	Exposure	Overall quality
Al Sulaiman, K. A.（2021）[8]	***		***	6
Allard L（2020）[65]	***		***	6
Anuk, A. T.（2021）[42]	***	*	***	7
Beigmohammadi, M. T.（2021）[77]	***	*	**	6
Dahan, S. (2020)[4]	***		***	6
Erol, S. A.（2021）[43]	***	*	***	7
Ersöz, A(2021)[9]	***		***	6
Lv, Y.（2021）[6]	***	*	***	7
Skalny, A. V.（2021）[16]	***	*	***	7
Tojo, K.（2021）[7]	***		**	5
Yağcı, S.（2021）[10]	***	*	***	7
Yasui, Y.（2020）[70]	****		***	7
Zeng, H. L.（2021）[22]	***		***	6
Zhou, C.（2020）[24]	***	*	***	7
Nai, A.（2021）[11]	****		***	7
Uta, M（2022）[17]	***	*	***	7
Bianconi, V（2022）[12]	***		***	6
Delaye, JB（2022）[18]	****	*	***	8
Zhao, K.（2020）[23]	***		***	6
Kronstein-Wiedemann, R (2022)[19]	****		***	7
Catherine, C（2021）[14]	***		***	6
Moreira, AC（2021）[20]	***		***	6
Ahmed, S (2021)[46]	***		***	6
Kilercik, M（2022）[15]	***	*	***	7
Ergin Tuncay, M (2022)[21]	****		***	7
Wu, C（2020）[47]	***		***	6
Deng, F (2020)[38]	***		***	6
Guan, X (2020)[48]	***		***	6
Tural Onur, S (2021) [49]	***		***	6
Branco,C.G.(2021)[39]	**	*	***	6
Lino, K (2021)[50]	***		***	6
Khamis, F (2021)[51]	***		***	6
Bozkurt, F.T. (2021)[78]	***	*	***	7
García-Gasalla, M (2021)[71]	***		***	6
Venter, C (2020)[44]	****	*	***	8
Rahman, M.A. (2021)[25]	****		***	7
Yardımcı, A.C. (2021)[52]	****		***	7
Kirtana, J.（2020）[67]	**	*	***	6
Martinez Mesa, A.(2021)[53]	****	*	***	8
Rasyid, H. (2021)[54]	***	*	***	7
Sukrisman, L. (2021)[68]	***		***	6
Zanella, A.（2021）[55]	***	*	***	7
Az, A. (2021)[30]	***		***	6
Burugu, H.R. (2020)[56]	***		***	6
Chakurkar, V.（2021）[13]	****	*	***	8
Rai, D. (2021)[57]	***	*	***	7
Aygun, H.（2021）[58]	***		***	6

San Segundo, D. (2021)[69]	****	*	***	8
Pujani, M. (2021)[59]	****	*	***	8
Haroun, R.A. (2021)[72]	**	**	***	7
Sukrisman, L. (2021)[79]	**	*	***	6
Chen,Q.（2020）[60]	***		***	6
Gayam, V.（2020）[40]	***	*	***	7
Ghweil, A.A. (2020)[31]	***	*	***	7
Ramadan, H.K.（2020）[32]	***		***	6
Zeng,Z.(2020)[41]	***		***	6
Yamamoto, A.（2021）[33]	***		***	6
Abdelhakam, D.A.（2021）[34]	***	*	***	7
Yousaf, M.N.（2022）[61]	***		***	6
Emsen, A.（2021）[35]	***	**	***	8
Doghish, A.S.（2021）[36]	***	**	***	8
Fei, F.（2020）[37]	***	*	***	7
Bats, M.L.（2021）[66]	***		***	6
Arshad, A.R. (2020)[62]	***		***	6
Aly, M.M. (2021)[26]	***	*	***	7
Garcia-Gasalla, M.（2022）[80]	***		***	6
Huang, H.（2021）[27]	***	*	***	7
Masetti, C.（2020）[63]	***	*	***	7
Nizami, D.J. (2021)[81]	***		***	6
Sana, A. (2022)[28]	***		***	6
Huang, C.Y. (2022)[29]	***	*	***	7
Marimuthu, A.K.（2021）[64]	***		***	6

The quality of studies was assessed by the Agency for Healthcare Research and Quality (ARHQ) methodology checklist. There are nine items about quality assessment, four items in Selection, two in Comparability and three in Exposure. For every item, "*" for Yes, blank for "No" or "Unclear". Therefore "*" means "Yes" for one item, "**" means "Yes" for two items and "***" means "Yes" for three items, blank for "No" or "Unclear" for all items. The score is the amount of "*" and the full score for the scale is nine points.
